# 3-Chloro­pyridin-2-amine

**DOI:** 10.1107/S1600536811013432

**Published:** 2011-04-16

**Authors:** Zhi-Nan Hu, Hui-Bin Yang, Huan Luo, Bin Li

**Affiliations:** a Department of Chemistry, Liaoning University, Shenyang 110036, People’s Republic of China; b Agrochemicals Division, Shenyang Research Institute of Chemical Industry, Shenyang 110021, People’s Republic of China

## Abstract

In the title compound, C_5_H_5_ClN_2_, a by-product in the synthesis of ethyl 2-(3-chloro­pyridin-2-yl)-5-oxopyrazolidine-3-carboxyl­ate, the amine groups form inter­molecular hydrogen-bonding associations with pyridine N-atom acceptors, giving centrosymmetric cyclic dimers. Short inter­molecular Cl⋯Cl inter­actions [3.278 (3) Å] also occur.

## Related literature

The title compound was isolated as a by-product in the preparation of ethyl 2-(3-chloro­pyridin-2-yl)-5-oxopyrazolidine-3-carboxyl­ate, an inter­mediate in the synthesis of the insecticide chlorantraniliprole (systematic name 3-bromo-*N*-[4-chloro-2-methyl-6-[(methyl­amino)carbon­yl]phen­yl]-1-(3-chloro-2-pyridin­yl)-1*H*-pyrazole-5-carboxamide), see: Lahm *et al.* (2005 ▶). For related structures, see: Chao *et al.* (1975 ▶); Anagnostis & Turnbull (1998 ▶); Hemamalini & Fun (2010 ▶).
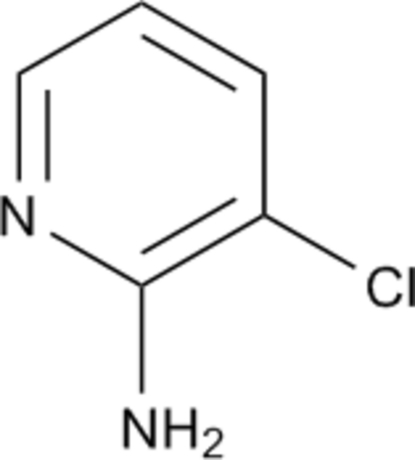


## Experimental

### 

#### Crystal data

C_5_H_5_ClN_2_
*M* *_r_* = 128.56
Monoclinic, 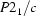

*a* = 11.149 (8) Å
*b* = 5.453 (4) Å
*c* = 9.844 (7) Å
β = 90.581 (12)°
*V* = 598.5 (7) Å^3^
*Z* = 4
Mo *K*α radiation
μ = 0.52 mm^−1^
*T* = 296 K
0.38 × 0.32 × 0.22 mm

#### Data collection

Bruker SMART CCD area-detector diffractometer
Absorption correction: multi-scan (*SADABS*; Bruker, 2001 ▶) *T*_min_ = 0.827, *T*_max_ = 0.894
2778 measured reflections
1057 independent reflections
867 reflections with *I* > 2σ(*I*)
*R*_int_ = 0.048

#### Refinement

*R*[*F* ^2^ > 2σ(*F* ^2^)] = 0.059
*wR*(*F* ^2^) = 0.182
*S* = 1.05
1057 reflections
73 parameters
H-atom parameters constrained
Δρ_max_ = 0.57 e Å^−3^
Δρ_min_ = −0.31 e Å^−3^

### 

Data collection: *SMART* (Bruker, 2001 ▶); cell refinement: *SAINT* (Bruker, 2001 ▶); data reduction: *SAINT*; program(s) used to solve structure: *SHELXS97* (Sheldrick, 2008 ▶); program(s) used to refine structure: *SHELXL97* (Sheldrick, 2008 ▶); molecular graphics: *SHELXTL* (Sheldrick, 2008 ▶); software used to prepare material for publication: *SHELXTL*.

## Supplementary Material

Crystal structure: contains datablocks I, global. DOI: 10.1107/S1600536811013432/zs2107sup1.cif

Structure factors: contains datablocks I. DOI: 10.1107/S1600536811013432/zs2107Isup2.hkl

Additional supplementary materials:  crystallographic information; 3D view; checkCIF report

## Figures and Tables

**Table 1:** Hydrogen-bond geometry (Å, °)

*D*—H⋯*A*	*D*—H	H⋯*A*	*D*⋯*A*	*D*—H⋯*A*
N2—H2*A*⋯N1^i^	0.86	2.22	3.051 (5)	162

Symmetry code: (i) 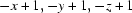.

**Fig. 1.**
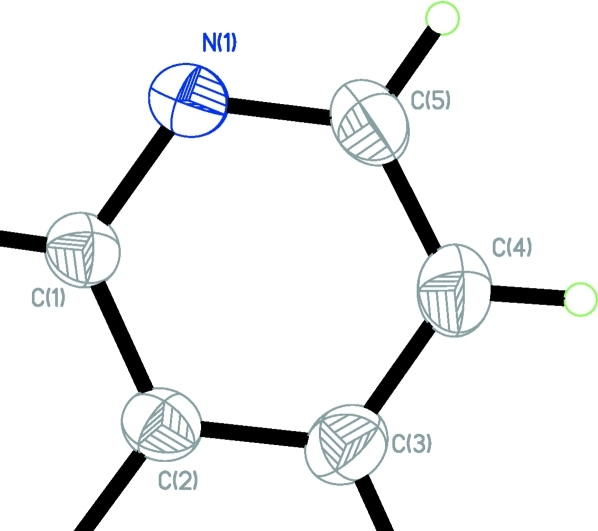
The molecular structure of (I), showing atom numbering scheme and 30% probability displacement ellipsoids.

**Fig. 2.**
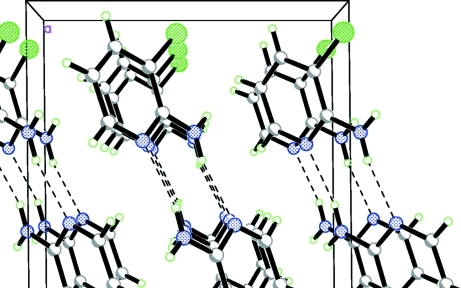
The packing of (I) in ther unit cell viewed down b, showing hydrogen-bonding interactions as dashed lines.

## supplementary crystallographic information

### Comment

The structures of salts of the halo-substituted aminopyridine, such as 2-amino-5-chloropyridine-fumaric acid (Hemamalini & Fun, 2010), 2-amino-3,5-dichloropyridinium chloride monohydrate (Anagnostis & Turnbull, 1998), are known but the the structure of 2-amino-3-chloropyridine is not known. This compound, C_5_H_5_Cl_1_N_2_ (I) was isolated as a by-product in the preparation of ethyl 2-(3-chloropyridin-2-yl)-5-oxopyrazolidine-3-carboxylate, an important intermediate in the synthesis of the insecticide chlorantraniliprole (3-bromo-N-[4-chloro-2-methyl-6-[(methylamino) carbonyl]phenyl]-1-(3-chloro-2-pyridinyl)-1H-pyrazole-5-carboxamide) (Lahm *et al.*, 2005).  In the structure of (I) (Fig. 1), intermolecular amine N—H···N_pyridine_ hydrogen-bonding interactions (Table 1) give centrosymmetric cyclic dimers (Fig. 2), similar to those found in the structure of 2-aminopyridine (Chao *et al.*, 1975). In (I) there is an intramolecular N—H···Cl interaction [3.001 (3) Å] while in the crystal structure there are also short Cl···Cl^ii^ interactions [3.278 (3) Å] [symmetry code: (ii) -*x* + 2, -*y*, -*z* + 1].

### Experimental

Sodium ethoxide (3.48 g, 50.4 mmol) and 150 ml of absolute ethanol was heated to reflux, after wich 6.80 g (47.4 mmol) of 3-chloro-2-hydrazinylpyridine was added and the mixture was allowed to reflux for 5 minutes. The slurry was then treated dropwise with 9.79 g (56.9 mmol) of diethyl maleate over a period of 5 minutes and the resulting solution was held at reflux for 10 minutes. After cooling to 338 K, the reaction mixture was treated with 5.0 ml (87.3 mmol) of glacial acetic acid. The mixture was diluted with 60 ml water and then cooled to room temperature, giving a precipitate which was isolated *via* filtration, and separated by column chromatography on silica gel (eluent: ethyl acetate/petroleum ether, 1:5). The title compound was obtained as a yellow solid (0.60 g, 8%) and recyrstallized from dichloromethane to afford colorless single crystals suitable for X-ray diffraction. Anal.: Calc. for C_5_H_5_Cl_1_N_2_: C, 46.47; H, 3.84; Cl, 27.96; N, 21.85%. Found: C, 46.71; H, 3.99; Cl, 27.58; N, 21.79. ^1^H NMR(CDCl_3_): 5.02(s,2*H*, NH_2_), 6.62(dd,1*H*, pyridine-H), 7.48(dd, 1*H*, pyridine-H), 7.98 (dd, 1*H*, pyridine-H).

### Refinement

Although all H atoms were visible in difference maps, they were placed in geometrically calculated positions, with N—H and C—H = o.86 and 0.93 Å respectively, and included in the final refinement in the riding model approximation, with *U*_iso_(H) = 1.2*U*_eq_(C).

### Crystal data

**Table:**

C_5_H_5_ClN_2_	*F*(000) = 264
*M**_r_* = 128.56	*D*_x_ = 1.427 Mg m^−^^3^
Monoclinic, *P*2_1_/*c*	Mo *K*α radiation, λ = 0.71073 Å
Hall symbol: -P 2ybc	Cell parameters from 1473 reflections
*a* = 11.149 (8) Å	θ = 3.7–27.2°
*b* = 5.453 (4) Å	µ = 0.52 mm^−^^1^
*c* = 9.844 (7) Å	*T* = 296 K
β = 90.581 (12)°	Block, yellow
*V* = 598.5 (7) Å^3^	0.38 × 0.32 × 0.22 mm
*Z* = 4	

### Data collection

**Table:**

Bruker SMART CCD area-detector diffractometer	1057 independent reflections
Radiation source: fine-focus sealed tube	867 reflections with *I* > 2σ(*I*)
graphite	*R*_int_ = 0.048
φ and ω scans	θ_max_ = 25.0°, θ_min_ = 1.8°
Absorption correction: multi-scan (*SADABS*; Bruker, 2001)	*h* = −13→11
*T*_min_ = 0.827, *T*_max_ = 0.894	*k* = −6→6
2778 measured reflections	*l* = −8→11

### Refinement

**Table:**

Refinement on *F*^2^	Primary atom site location: structure-invariant direct methods
Least-squares matrix: full	Secondary atom site location: difference Fourier map
*R*[*F*^2^ > 2σ(*F*^2^)] = 0.059	Hydrogen site location: inferred from neighbouring sites
*wR*(*F*^2^) = 0.182	H-atom parameters constrained
*S* = 1.05	*w* = 1/[σ^2^(*F*_o_^2^) + (0.1147*P*)^2^ + 0.2179*P*] where *P* = (*F*_o_^2^ + 2*F*_c_^2^)/3
1057 reflections	(Δ/σ)_max_ < 0.001
73 parameters	Δρ_max_ = 0.57 e Å^−^^3^
0 restraints	Δρ_min_ = −0.31 e Å^−^^3^

### Special details

**Table:**

Geometry. All e.s.d.'s (except the e.s.d. in the dihedral angle between two l.s. planes) are estimated using the full covariance matrix. The cell e.s.d.'s are taken into account individually in the estimation of e.s.d.'s in distances, angles and torsion angles; correlations between e.s.d.'s in cell parameters are only used when they are defined by crystal symmetry. An approximate (isotropic) treatment of cell e.s.d.'s is used for estimating e.s.d.'s involving l.s. planes.
Refinement. Refinement of *F*^2^ against ALL reflections. The weighted *R*-factor *wR* and goodness of fit *S* are based on *F*^2^, conventional *R*-factors *R* are based on *F*, with *F* set to zero for negative *F*^2^. The threshold expression of *F*^2^ > σ(*F*^2^) is used only for calculating *R*-factors(gt) *etc*. and is not relevant to the choice of reflections for refinement. *R*-factors based on *F*^2^ are statistically about twice as large as those based on *F*, and *R*- factors based on ALL data will be even larger.

### Fractional atomic coordinates and isotropic or equivalent isotropic displacement parameters (Å^2^)

**Table:**

	*x*	*y*	*z*	*U*_iso_*/*U*_eq_	
Cl1	0.89884 (8)	0.20978 (18)	0.46576 (10)	0.0821 (5)	
N1	0.6085 (2)	0.5920 (5)	0.3676 (2)	0.0597 (7)	
N2	0.6357 (3)	0.2720 (5)	0.5172 (3)	0.0716 (8)	
H2A	0.5613	0.2836	0.5385	0.086*	
H2B	0.6804	0.1628	0.5553	0.086*	
C1	0.6825 (2)	0.4252 (5)	0.4237 (3)	0.0505 (7)	
C2	0.8035 (2)	0.4167 (5)	0.3855 (3)	0.0535 (7)	
C3	0.8465 (3)	0.5728 (6)	0.2897 (3)	0.0635 (8)	
H3	0.9266	0.5667	0.2645	0.076*	
C4	0.7692 (3)	0.7404 (7)	0.2306 (3)	0.0725 (10)	
H4	0.7955	0.8481	0.1640	0.087*	
C5	0.6520 (3)	0.7431 (6)	0.2735 (4)	0.0698 (9)	
H5	0.6000	0.8571	0.2345	0.084*	

### Atomic displacement parameters (Å^2^)

**Table:**

	*U*^11^	*U*^22^	*U*^33^	*U*^12^	*U*^13^	*U*^23^
Cl1	0.0717 (7)	0.0833 (7)	0.0913 (8)	0.0322 (4)	0.0101 (5)	0.0145 (4)
N1	0.0545 (13)	0.0566 (14)	0.0681 (15)	0.0068 (11)	0.0018 (10)	0.0047 (11)
N2	0.0674 (16)	0.0602 (16)	0.088 (2)	0.0131 (12)	0.0194 (14)	0.0196 (13)
C1	0.0572 (14)	0.0411 (13)	0.0533 (15)	0.0036 (11)	0.0026 (11)	−0.0039 (11)
C2	0.0558 (15)	0.0510 (15)	0.0537 (15)	0.0099 (11)	0.0019 (11)	−0.0057 (12)
C3	0.0561 (15)	0.076 (2)	0.0583 (17)	−0.0019 (14)	0.0070 (13)	0.0002 (14)
C4	0.077 (2)	0.074 (2)	0.067 (2)	−0.0049 (15)	0.0047 (17)	0.0178 (15)
C5	0.073 (2)	0.0617 (19)	0.074 (2)	0.0057 (14)	−0.0054 (16)	0.0155 (15)

### Geometric parameters (Å, °)

**Table:**

Cl1—C2	1.735 (3)	C2—C3	1.361 (4)
N1—C5	1.334 (4)	C3—C4	1.380 (4)
N1—C1	1.344 (4)	C3—H3	0.9300
N2—C1	1.351 (4)	C4—C5	1.378 (5)
N2—H2A	0.8600	C4—H4	0.9300
N2—H2B	0.8600	C5—H5	0.9300
C1—C2	1.405 (4)		
			
C5—N1—C1	118.5 (3)	C2—C3—C4	118.9 (3)
C1—N2—H2A	120.0	C2—C3—H3	120.6
C1—N2—H2B	120.0	C4—C3—H3	120.6
H2A—N2—H2B	120.0	C5—C4—C3	117.9 (3)
N1—C1—N2	117.3 (3)	C5—C4—H4	121.0
N1—C1—C2	120.0 (2)	C3—C4—H4	121.0
N2—C1—C2	122.7 (2)	N1—C5—C4	124.0 (3)
C3—C2—C1	120.7 (3)	N1—C5—H5	118.0
C3—C2—Cl1	120.2 (2)	C4—C5—H5	118.0
C1—C2—Cl1	119.0 (2)		
			
C5—N1—C1—N2	−179.0 (3)	C1—C2—C3—C4	0.1 (5)
C5—N1—C1—C2	1.5 (4)	Cl1—C2—C3—C4	−178.0 (2)
N1—C1—C2—C3	−1.3 (4)	C2—C3—C4—C5	0.9 (5)
N2—C1—C2—C3	179.2 (3)	C1—N1—C5—C4	−0.6 (5)
N1—C1—C2—Cl1	176.8 (2)	C3—C4—C5—N1	−0.7 (5)
N2—C1—C2—Cl1	−2.6 (4)		

### Hydrogen-bond geometry (Å, °)

**Table:**

*D*—H···*A*	*D*—H	H···*A*	*D*···*A*	*D*—H···*A*
N2—H2A···N1^i^	0.86	2.22	3.051 (5)	162
N2—H2B···Cl1	0.86	2.61	3.001 (4)	109

Symmetry codes: (i) −*x*+1, −*y*+1, −*z*+1.
